# Designing for Psychological Change: Individuals’ Reward and Cost Valuations in Weight Management

**DOI:** 10.2196/jmir.3009

**Published:** 2014-06-26

**Authors:** Anne Hsu, Ann Blandford

**Affiliations:** ^1^School of Electronic Engineering and Computer ScienceQueen Mary, University of LondonLondonUnited Kingdom; ^2^UCL Interaction CentreUniversity College LondonLondonUnited Kingdom

**Keywords:** design, human-centered computing, behavior, psychology, interaction design process and methods, weight loss, health promotion

## Abstract

**Background:**

Knowledge of the psychological constructs that underlie behavior offers valuable design opportunities for persuasive systems. We use the decision theory, which describes how behavior is underpinned by reward-cost valuations, as a framework for investigating such psychological constructs to deliver design objectives for weight management technologies.

**Objective:**

We applied a decision theory–based analysis in the domain of weight management to understand the rewards and costs that surround individuals’ weight management behaviors, with the aim of uncovering design opportunities for weight management technologies.

**Methods:**

We conducted qualitative interviews with 15 participants who were or had been trying to lose weight. Thematic analysis was used to extract themes that covered the rewards and costs surrounding weight management behaviors. We supplemented our qualitative study with a quantitative survey of 100 respondents investigating the extent to which they agreed with statements reflecting themes from the qualitative study.

**Results:**

The primary obstacles to weight management were the rewards associated with unhealthy choices, such as the pleasures of unhealthy foods and unrestricted consumption in social situations, and the significant efforts required to change habits, plan, and exercise. Psychological constructs that supported positive weight management included feeling good after making healthy choices, being good to oneself, experiencing healthy yet still delicious foods, and receiving social support and encouraging messages (although opinions about encouraging messages was mixed).

**Conclusions:**

A rewards-costs driven enquiry revealed a wide range of psychological constructs that contribute to discouraging and supporting weight management. The constructs extracted from our qualitative study were verified by our quantitative survey, in which the majority of respondents also reported similar thoughts and feelings. This understanding of the rewards and costs surrounding weight management offers a range of new opportunities for the design of weight management technologies that enhance the encouraging factors and alleviate the discouraging ones.

## Introduction

### Background

Obesity is one of the most serious world health problems of the 21st century. Excessive weight has been identified as the cause of many medical conditions, which have been estimated to cost $147 billion per year in the United States alone [1]. In response to this problem, a rising number of weight management technologies are being developed. Because thoughts and feelings are fundamental drivers of behavior, it is important for persuasive technologies to be informed by an understanding of the psychological underpinnings of behavior. Indeed, general frameworks for persuasive systems design have highlighted the importance of understanding the motivational landscape [2,3], or more generally, the *persuasion context*, which includes the thoughts, feelings, and attitudes of the individual being persuaded [4,5].

Psychological understanding can inform persuasive systems design at varying levels of specificity. At a macro level, there are context-general psychological principles, such as theories of behavior change. These theories describe general psychological principles and offer general strategies for influencing behavior. These include goal setting theory [6], transtheoretical model of stages of behavioral change [7], and social cognitive theory [8]. Such general behavior change theories have recently begun to influence persuasive systems design [9]. In addition to aiming to change behavior directly, persuasive technologies can also effect change by targeting the psychological constructs that underlie behaviors. Indeed, long-term behavioral changes will be sustained only when the relevant underlying psychological constructs are altered [4,5,10]. Therefore, in order to influence the psychological constructs that underpin behaviors, it is important to understand what these constructs are within a specific persuasion context. Unlike general behavior change theories, these psychological constructs are not domain general but instead capture the particular thoughts, feelings, and attitudes that surround behaviors relevant to a specific context, for example, weight loss, smoking cessation, or road safety. Thus, whereas general theories of behavior change provide the designer with strategies of *how to change,* an understanding of specific psychological constructs helps inform the designer of *what to change.* The latter has not been highlighted in the discussion of persuasive systems design. In the current work, we propose that the considerations of rewards and costs in decision theory [10] offer an appropriate framework for both extracting key context-specific psychological constructs and also for specifying how to translate knowledge of these constructs into design targets for persuasive systems.

### Persuasive Systems Design

Persuasive systems effect change through two primary (non-mutually exclusive) avenues: shaping external behaviors or internal attitudes. The overwhelming majority of existing persuasive technologies focus on external behaviors [11], for example, a tracker for calorie consumption and exercise [9,11-14]. This is because external behaviors are more tangible, measurable, and thus easier targets for change. However, the distinction between influencing behavior vs thought is blurred: often influencing behavior also promotes new thoughts and awareness. For example, exercise and calorie trackers increase user awareness of their activities and intake. There has been a recent increase in persuasive technologies designed for psychological influence by encouraging reflection and awareness about health-related choices. These include text message-based interventions that prompt self-reflection [15], a visual garden display to encourage emotional connection to one’s levels of physical activity [16,17], open-ended platforms for documenting and sharing health decisions, and a shared visual journal for diet and exercise [14].

It is important for persuasive systems to change attitudes as well as behavior because attitudes and preferences underpin human choices and behavior [4,5,10]. In order to produce more robust behavioral changes, recent persuasive health technologies have started to shape people’s psychological responses, attitudes, and preferences by applying theories and strategies from psychology and health research [9,11,18-20]. The pervasive quality of many technological systems, such as mobile technologies, means that they are particularly well positioned to deliver interventions that target psychological processes. These advantages of pervasive technologies are summarized through a description of the many roles that such technologies play [21]: a device of “kairos” that is always near at hand, the role of a concierge that offers guidance and information at the moment of need, a personal coach that can track personal goals and context, and a jester that can promote fun interactions (both personal and social). These properties of technological systems make them well suited for modifying psychology because they can evoke new thought processes and emotions in a timely fashion, customized to the persuasion context. For example, technologies can be synchronized with electronic diaries or sensing technologies [22] to know when contexts that prompt salient psychological states will occur. Finally, thoughts, feelings, and attitudes are highly personal, and technology-based systems have the potential to deliver personalized interventions that cater to this individual variation.

While knowledge of general context-general psychological principles is highly useful for persuasive design, it is also important to understand the specific psychological constructs that underlie the behaviors in a particular design context. Persuasive technologies can then aim to alleviate the psychological constructs that discourage and enhance the ones that encourage. We propose that decision theory provides a useful starting point for investigating these psychological constructs because its framework of reward-cost considerations naturally defines clear design objectives for persuasive systems. We provide a brief overview of decision theory below.

### Decision Theory and Reward-Cost Considerations

The term “decision theory” has been applied to a wide variety of models describing how people make choices that give rise to behavior. At one extreme are economics-inspired models of rational agents that calculate the individual utilities associated with all attributes of choice options and then maximize summed utility. More psychologically realistic models highlight the context-dependent nature of attribute valuations [23], with some models depicting choices as entirely dependent on contextual comparisons [24], or “one shot” reasoning informed by heuristics and biases [23]. From a more local perspective, a large body of recent neuroscience research [10,25] has depicted decision making as follows: Behavior is characterized as a choice among possible behavior options. The mind associates rewards and costs with each behavioral option, and the most rewarding options are most likely to be chosen. In support of this, neuroimaging and neurophysiology studies have found that before a choice is made, each behavioral option is associated with a *decision value* [10], that is, a signal in the brain that represents a mental forecast of the option’s hedonic impact (the forecasted pleasure to be gained from the option). It is suggested that the brain computes decision values by summing up the predicted rewards and costs associated with each option [10,25]. A comparison is then made of the relative decision values among behavioral options, and behaviors that are assigned higher decision values are more likely to be chosen. We emphasize that decision theory focuses on *hedonic* rewards (eg, pleasure, social value, comfort) and costs (eg, pain, effort, discomfort, embarrassment). This means, for example, that a choice that has monetary cost is less desirable only because of its associated “emotional cost” of the negative feelings (eg, guilt, pain, worry) of losing money.

The decision theoretic framework we have described is broad and is consistent with a wide range of psychological-level models of decision making [25]. For example, the types of attributes considered and how they are valued have been shown to be highly modulated by context and attention [26,27], which is consistent with psychological models. Also, decision values may be informed by only a single attribute (consistent with one-shot heuristic approaches to decision making), or multiple attributes [25]. This framework is also not limited to conscious attitudes. For example, unconscious saccades in eye movements have also been described using reward-cost valuations of decision theory [24,28]. However, for the purpose of persuasive systems design, it is usually most practical to focus on the conscious attitudes that can be elicited from the user.

A key feature of reward-cost considerations, highly relevant for weight loss, is delayed discounting, where it has been observed that delayed consequences are substantially less influential than immediate ones [29]. Many studies have shown that decision values in the brain are significantly more influenced by rewards and costs that will be experienced immediately rather than far off in the future [25]. The decreased value of delayed rewards lies at the heart of most self-control struggles [30] and is highly relevant to weight management because weight change mostly occurs over long time frames. Thus weight-relevant choices (and all behavior choices) will be more influenced by immediate feelings than long-term consequences. Immediate feelings can consist of feelings of physical sensations as well as psychological feelings of satisfaction. Therefore, when a dieter chooses a healthy snack option, his or her decision values are usually less influenced by distant attributes, such as the eventual weight loss, and more influenced immediate attributes, such as the feeling of satisfaction of having made a healthy choice. This is important to bear in mind when designing persuasive systems. One possible role for persuasive systems is to transform long-term consequences into more immediate feelings (eg, show pictures of additional fat gained after a big meal).

Recent promising research shows that rewards and cost valuations can be altered to shift preferences. On a neural level, it has been found that reward associations for healthy options can be enhanced to result in preferences for healthier choices. Prompting a focus on health rather than taste increases the brain’s decision values (ie, preferences) and choices, for healthier foods [27]. Prompting thoughts about future events has been shown to reduce delayed discounting and increase decision values (ie, preference) for delayed rewards [26]. Similar behavioral research has also found that preferences can be shifted towards healthier choices. Emphasizing enjoyable experiences associated with consumption of healthy foods results in these foods being chosen more often in later meals [31]. There has also been behavioral research showing that preferences for unhealthy eating behaviors can be decreased. Use of visuospatial tasks and imagery have been shown to reduce food craving levels [32].

### Applying Decision Theory to Inform Persuasive Design

We propose that the broad framework of decision theory, which considers the rewards and costs associated with behavioral options, can be used to inform persuasive systems design. This is key to behavior change as deeply rooted behaviors are likely to have strong emotional components. The framework can be used as follows. First, the designer specifies the desired and undesired behaviors within the persuasive design context. The designer then aims to identify the rewards and costs associated with these desirable and undesirable behaviors. After the rewards and costs have been identified, decision theory specifies four ways in which persuasive systems can encourage desired behaviors: (1) increase the rewards associated with desired behaviors, (2) decrease the costs associated with desired behaviors, (3) decrease the rewards associated with undesired behaviors, and (4) increase the costs associated with the undesired behaviors.

### Why Decision Theory?

Decision theory is a low-level approach to understanding behavior that underpins a large family of motivational theories, such as Theory of Planned Behavior [33], Theory of Interpersonal Behavior [34], and Theory of Normative Social Behavior [34]. Different theories of behavior capture human motivation at varying levels of abstraction, and with emphasis on different features of behavior [35]. While all levels of motivational theory can be useful for persuasive systems design, we propose that the decision theoretic approach of considering rewards and costs are at an appropriate level of abstraction to serve as a starting point for investigating the relevant psychological constructs involved. An investigation of rewards and costs over a range of weight management contexts imposes minimal assumptions and offers the most open-ended foundation for discovering design opportunities.

### Aims of Current Work

The aim of the work reported here is two-fold: (1) to test whether the decision theory framework of reward-cost valuations can be used to reveal the psychological constructs that underlie behavior and thus inform design of weight management systems, and (2) to better understand the persuasion context relevant for weight management, that is, what thoughts, feelings, and contexts encourage or discourage people’s weight management efforts.

We present results from an in-depth interview study aimed at revealing the rewards and costs associated with weight management efforts. We asked individuals to share the thoughts, feelings, and contexts that surrounded their weight loss efforts. We hypothesized that (1) these interviews would reveal some key rewards and costs relevant to weight management choices, and (2) understanding of these rewards and costs would yield potential design objectives for weight management technologies. We organized the presentation of our interview results into rewards and costs surrounding behaviors that discourage versus encourage weight management. Following our qualitative study, we conducted a Web-based survey to understand the extent to which the themes from our interviews were experienced over a larger population. Finally, we discuss the implications of our results for the design of weight management technologies.

## Methods

### Summary

We sought to understand the rewards and costs associated with both negative and positive weight management behaviors. To do so, we first conducted a qualitative study consisting of semistructured interviews that aimed to identify the rewards and costs surrounding weight management behaviors. We then supplemented our qualitative study with a quantitative survey to assess the extent to which the themes we extracted from our qualitative study were experienced over a larger population.

### Methods for Qualitative Study

#### Qualitative Study Procedure

In our qualitative study, we asked individuals about the thoughts, feelings, and contexts that surrounded behaviors that either discouraged or encouraged weight loss efforts. Semistructured interviews were conducted lasting 45-60 minutes. Questions were structured around the thoughts, feelings, actions, and situations that surrounded either successful or unsuccessful weight loss behaviors (see Multimedia Appendix 1). Participants were also asked to describe specific instances where they were either unsuccessful or successful in pursuing their weight goals and asked to elaborate on the surrounding thoughts, feelings, and circumstances. We also asked participants general weight management questions such as weight history, progress, peer involvement, and strategies used. All participants were provided with an information sheet and a consent form to sign and return to the researcher. The study materials, data collection, and methods of data storage were in accordance with human subjects guidelines set and approved by the Queen Mary University of London ethics committee.

#### Qualitative Study Recruitment

We interviewed 15 individuals with current or previous experience of trying to lose weight. We included participants who had previously tried to lose weight so that we could capture a wider range of experiences, such as having already successfully maintained lost weight or unsuccessfully given up diet efforts. We focused on eating habits and, to a lesser extent, exercise. Interview recruitment emails were sent out through several departments within a UK university. The emails invited individuals and their friends and families who have been or are engaged in weight control efforts to talk about their experiences of weight management. The recruitment resulted in 15 respondents, 12 of whom were female. All participants recruited were actively engaged in some level of weight management activities at the time of the study, that is, they were either trying to lose weight (n=13) or just trying to maintain their current weight, having attempted weight loss in the past, either successfully (n=1) or unsuccessfully (n=1). All participants were managing their weight through some combination of watching their diet and exercise. Ages ranged from 27-68 years with a median age of 35. Body Mass Index (BMI) ranged from 21.6 to 31.2 with a median of 23.4. (We note that while all of our participants either were trying to lose weight or have lost weight previously, only 5 of our participants classified as overweight, with BMI>25, and only one those qualified as obese, with BMI>30.) Table 1 shows the demographics of gender, age, BMI, and location for each participant. Of the 15 participants, 13 were based in the United Kingdom and 2 were based in the United States (and were interviewed on Skype). They had all completed a university degree and spanned a range of professions including human resources, marketing, interior design, engineering, science, journalism, publishing, and accounting. Each participant was recompensed for their time through the award of a £6/US$9 gift voucher.

**Table 1:** Participant demographics and BMI.

	P1	P2	P3	P4	P5	P6	P7	P8	P9	P10	P11	P12	P13	P14	P15
Gender	F	F	M	F	F	F	F	M|	M	F	F	F	F	F	F
Age	35	30	36	39	35	43	27	42	68	30	35	56	36	33	32
BMI	22.1	22.6	22.3	25.6	26.4	27.5	23.8	22.8	24.8	31.2	23.2	21.6	21.9	23.4	27.6

#### Qualitative Study Analysis

Each interview was audio recorded and transcribed. We then employed inductive thematic analysis as described by Braun and Clarke [36]. All stages of analysis were conducted by the first author, and method and findings were discussed with the co-author at intervals.

Transcripts were initially analyzed for patterns across all interviews to extract themes about constructs that either discouraged or encouraged weight management. The data were repeatedly revisited to consolidate the main themes that were present across interviews. Once consistent themes were identified, all transcripts were re-analyzed, and all data extracts relevant to each theme were coded.

### Methods for Quantitative Study

#### Quantitative Study Procedure

We supplemented our qualitative study with a quantitative survey to assess the extent to which the results we found in our qualitative study were valid across a larger population. While the interviews were conducted with highly educated, primarily UK-based participants, the survey was held among the general American public. Our survey asked respondents to assess agreement with statements that represented the constructs and themes we uncovered from our qualitative study. We asked participants to rate their level of agreement on a 7-point multiple choice Likert scale ranging from Strongly Agree to Strongly Disagree. We also asked participants their age, BMI, and whether they were trying to lose weight, maintain weight, gain weight, or did not think about their weight.

#### Quantitative Study Recruitment

Participants were 100 US-based adults recruited using Amazon Mechanical Turk. To minimize droid respondents, only participants with an approval rating of 95% or higher on Amazon Turk were allowed. They were compensated US$1 for completing our survey. Amazon Turk does not allow participants to respond twice to the same survey. Participants were provided with an information screen and a consent screen on which they had to click the “I agree” button in order to demonstrate consent for participation in the online study. The study materials, data collection, and methods of data storage were in accordance with human subjects guidelines set and approved by the Queen Mary University of London ethics committee.

## Results

### Qualitative Study Results

#### Summary

Our interviews revealed a wide range of rewards and costs that were associated with both negative and positive weight management behaviors. This supports the relevance of this line of enquiry for informing the design of persuasive systems. We first describe the psychological constructs that discouraged weight management, which consist of the need to restrict rewards (positive feelings and experiences) associated with unhealthy behaviors, and the direct costs (negative feelings and experiences) associated with weight management efforts. Next we describe the psychological constructs that surrounded positive weight management behavior, which consists of emphasizing the rewards associated with weight management efforts and the costs associated with unhealthy behaviors.

#### Discouraging Weight Loss Efforts

##### Overview

A key challenge to weight loss was the effort needed to restrict pleasure and change habits, overcome social pressures, plan ahead, and exercise. Participants also spoke of the loss of rewards associated with restricting consumption of unhealthy foods and inability to participate freely in social situations with food and drinks. Furthermore, participants felt daunted by thoughts that such weight loss efforts need to be sustained long term. Many participants voiced frustration over the fact that weight loss was a slow and unsteady process. Finally, several participants also mentioned negative associations with the general concept of dieting. We provide more details on these themes below.

##### Effort of Restricting Pleasure and Changing Habit

Most participants mentioned that modifying diet for weight loss was difficult. Circumstances where healthy eating was generally reported as being difficult included when bored, tired, stressed, busy, hungry, unhappy, and also when food was immediately present. For many, mental space and time were required for weight loss efforts. Some participants reported feeling that the effort was not worth it when life was more strenuous: “It’s quite hard sometimes. You’ve really got to put so much energy into it and it takes up so much time and space in your life. (In the past) I just didn’t want it enough” [P5] and “At the moment I just don’t have headspace to be as disciplined as I’d like with my food” [P15].

##### Loss of Pleasure

Most participants described food as being a significant source of pleasure or reward, which they lose out on when trying to lose weight, for example, “I enjoy food a lot, so I’ll eat if I’m not hungry…I don’t like to deny myself things I enjoy” [P2].

##### Restricting Social Participation

The role of social influence in weight control has been widely acknowledged [37]. Unsurprisingly, many of our participants mentioned that social pressures made dieting difficult. About half of the participants felt that making healthy diet choices often required going against social expectations when eating among others: “I went to pub…I realized I would feel like a bit of an outcast if I had something healthy” [P11] and “There’s a social pressure to everyone doing and enjoying the same type of activity” [P13].

##### Planning

About half of our participants mentioned that weight loss involved significant planning, which required mental space and time. These include planning healthy meals and snacks, workout schedules, and how to handle upcoming situations where there may be temptation to eat or drink too much, for example, “My biggest thing is you’ve got to plan the week ahead, you can’t just wake up and think what exercise do I do today?” [P5].

##### Long-Term Commitment Is Daunting

Some participants mentioned being put off by the knowledge that weight loss efforts needed to be sustained long term. Several participants had already experienced regaining weight lost on previous diets. The need for long-term behavior changes increases the perceived costs of weight loss efforts. For P10 and P15, the likelihood of regaining the weight afterwards made weight loss efforts feel less worthwhile: “The hardest thing is kind of recognizing it’s a shift in lifestyle rather than something you do for a little while and give up on” [P10] and “It’s also the long-term thing that looms over me…you’ve got to do that for the rest of your life. Otherwise there’s no point” [P15]. However, we note that this perception was not the case for all participants, and there were other participants (P8 and P13) who mentioned that maintenance was much easier than trying to lose.

##### Frustration Over Slow, Unsteady Progress

Several participants felt frustrated that they could not immediately experience rewards for their healthy behaviors. They felt frustrated at the slow and unsteady nature of weight loss. P7 described the need to focus on motivations other than weight loss: “Don’t want to be reminded what weight I am. That’s not motivating! Because it doesn’t change quickly enough, you just don’t feel any progress from it” [P7].

P5 and P13 both voiced frustration over the fact that consistent adherence to diet and exercise regimens did not always translate to regular weight loss. They both discussed how much easier it would be if weight just decreased at a steady rate, for example, “If I’ve spent several days exercising like crazy, not eating the things and not drinking...and nothing is happening, I’m angry…Sometimes you can eat a load of food and lose weight. I think in a way we’d prefer it to be a bit fairer” [P5].

P13 expressed particular frustration over the simplistic nature of calorie and fitness tracking applications, which implicitly assume that calorie deficits readily translate to weight loss, which was not the case for her:

Another thing about the technology, because you keep putting the calories in, because it’s so quantitative I feel like well “shouldn’t I be losing the weight?”, and it isn’t necessarily that way, a calorie in isn’t a calorie out, there is so much else going on in the physiology, so actually just seeing the quantitative representation of what you’re eating, vs how much you should be losing weight, it’s dispiriting.

P13

##### Notion of Diet Is Unappealing

Many participants expressed negative associations (ie, costs associated) with the concept of “being on a diet”. They expressed dislike of overemphasizing the importance of dieting and/or disliked thinking of themselves as formally being on a diet. P11 and P15 mentioned that too much focus on dieting felt superficial. P3 described dieting as pretentious: “(Dieting) feels prissy, feels pretentious. I’m a man, I have a big appetite, I do what I want when I want” [P3].

P9 and P12 felt that a focus on “dieting” would be ineffectual and even counterproductive: “If you asked me if I were on a diet, I’d say no. (To me, dieting is) this sort of deliberate pushing yourself kind of thing, and I’d say I’m not doing that, I don’t know that it would work for me” [P9] and “I’ve found that worrying about weight per se is very counterproductive, screws me up and focuses on the wrong things” [P12].

#### Encouraging Weight Loss Efforts

##### Overview

Our interviews showed that there are a variety of rewards associated with weight management. These included feeling good after making healthy choices, being good to oneself, social support, and receiving encouraging messages. (However, there were significant individual differences in response to social support and encouraging messages, which we describe below.) All participants mentioned the importance of personalization in order to find strategies that were easiest and most effective for the individual. Several participants noted that weight loss efforts eased with time. Several participants spoke of making healthy choices easier by emphasizing the costs associated with unhealthy choices. We discuss these constructs that encouraged weight loss–promoting behavior in detail below.

##### Personalized Strategies for Reducing Effort

Many participants spoke of using personally tailored strategies to reduce the costs of their weight loss efforts. P2 and P5 found increased exercise most helpful for weight loss. They found exercise enjoyable and easier than restricting food. P4 had found a successful diet that consisted of low carbohydrate breakfast and lunch and no restrictions for dinner. She spoke of how this diet was surprisingly very easy to stick to because it was extremely well suited to her because she enjoyed eating meat. P9 described the strategy he adopted to fit around his unusually variable schedule, which included commuting between different cities. This erratic schedule prevented him from being able to plan ahead easily, and instead he looked to being “opportunistic” and taking advantage of circumstances in the moment, where it seemed easy to eat lighter meals rather than planning food restriction ahead of time:

I have to be opportunistic, I don’t think it would work if I was marking my diary ahead of time…it might just feel wrong that day. Or I might feel hungry. But on the other hand, if I’ve met my son for lunch and we had a small meal out, then I can say that’s my main meal then I can be opportunistic, that’s relatively easy to do.

P9

P11 talked about how she would consciously slow down her thinking to consider what she really wanted/needed to consume rather than reacting automatically to grab more unhealthy choices. She discovered that if she was mindful about what she really wanted, often she did not actually crave unhealthy foods after all and would prefer foods such as tea and salads over wine and cakes.

All participants acknowledged the importance of finding the right strategies for them. They used such phrases as “everybody’s different”, “I know other people who (are different)”, and “you have to find what works for you”. Across the interviews, personal physiologies, life circumstances, tastes, and preferences varied widely. Indeed, research has shown that individuals can have very different physiological responses to the same diet and exercise routines [38,39]. For example, people varied widely in terms of how their bodies responded to exercise and how their guts responded to foods. This variation was evident in our participants’ experiences. For example, in contrast to P5 and P13, who experienced significant periods of weight stagnation despite adhering to diet and exercise, P9 was surprised at how immediate his weight loss was—he said every time he ate lightly for one meal the night before, he could see measurable changes on the scale almost the next morning. Similarly P10 mentioned that significant weight loss happened for her only when she drastically reduced sodium intake, a phenomenon that no other participants mentioned. Variation in participants’ personal preferences obviously affected the appropriateness of particular weight loss strategies. P13 talked about how a high protein diet was difficult because she did not enjoy eating meat, in contrast to the success of P4 on such a diet. P1 spoke of how calorie restriction was far more effective than exercise, which is in contrast to the experiences of P2 and P5.

##### Easier With Time

Many participants mentioned that their weight loss efforts became easier with time: “I found it was harder for first couple weeks, and then it just became a habit to track the calories and not eat. You got used to feeling hungry” [P8].

##### Good Feelings, Pride, and Control

Most participants enjoyed positive feelings as a result of engaging in healthy behaviors. They mentioned feeling pleased, good, or happy after engaging in behavior that promoted weight loss. In particular, 6 participants mentioned enjoying the feeling of control and empowerment: “I feel quite empowered, feel like I own my weight, it’s going the correct direction” [P5] and “I’m focused, I have the energy for it, and it feels good to say you know what I’m not going to have that, I’m going to have the good thing” [P15].

Several participants mentioned feeling proud after making weight loss promoting choices, for example, “I feel very proud of myself. This is how I should be, I should be strong, I don’t really need this (cake)” [P6].

Conversely, when participants were asked how they felt immediately after having indulged in unhealthy behaviors, most reported that they enjoyed the indulgence during consumption, but the good feelings usually did not last long. Afterwards, some experienced negative feelings such as guilt or disappointment with self, or felt a bit “down”. Most participants did intellectually acknowledge the unhealthiness of their choice immediately after consumption. For example, P2 felt “naughty, that you’ve let yourself down. You enjoyed it, but you feel like you’ve cheated”.

##### Realism

Several participants mentioned that reminding themselves to be “realistic” about the costs of unhealthy behaviors helped encourage healthy choices at moments of temptation. These thoughts included reminders of the reality of the situation of being overweight and the reality of the negative consequences of consuming excessive/unhealthy foods:

I’ve actually had to say to myself in the past “it doesn’t matter what you say something is…You can pretend you have only drunk half a cup of milk but if you’ve drunk a whole cup that’s what’s going to have the impact”.

P5

I think to be very real, “think about it, once you eat it it’s going to be hard, and you know once you gain weight you will feel low esteem (and) sad”…I think it’s the reality that makes me think I don’t want this for myself anymore. Let’s move on, let’s not eat cake then.

P6

##### Being Good to Oneself

Many participants mentioned concepts relating to being good to oneself as part of successful weight management. These included being kind to oneself, framing weight loss as a process of self-care, reassuring oneself that one is not being deprived and that healthy choices will lead to greater happiness, and savoring the pleasurable aspects of healthy choices. P5 and P10 spoke of how developing an attitude of self-kindness and forgiveness helped facilitate getting back on track after stints of excessive, unhealthy consumption: “I’m being a lot gentler with myself these day. I’m not telling myself off and being so angry with myself. So I think it’s about forgiving myself for the mistakes rather than punishing myself” [P5] and “It’s recognizing some days when all I want to eat is pizza and that’s ok, and that doesn’t ruin all the rest of the eating, and forgiving myself for the splurges and going on like I wanted to” [P10].

P10 also spoke of how recognizing that exercise and healthier eating plays into the larger purpose of general self-care and improving well-being helped her make healthier choices: “One friend said maybe the reason you’re feeling crummy is you haven’t had the time to [eat healthily] so it was kind of a self-care thing as well” [P10].

Three participants reminded themselves that healthier choices did not mean deprivation, that this was not their last opportunity to eat delicious foods and that pleasurable foods were still available to them, if in moderation, for example, “I’d think ‘No, that’s hardly the last piece of delicious chocolate you’ll ever have in your life’” [P1].

P11 spoke of changing her mindset to recognize that by restricting treats, she was not depriving herself of tasty experiences, but instead was actually allowing herself greater overall pleasure because foods tasted better when eaten less often. Many participants mentioned relishing delicious healthy foods as well as small portions of rich foods as part of successful diet strategies. This appears to affirm the existence of reward and pleasure, even during the restrictive process of weight loss.

##### Social Support

There was great individual variation in terms of preferred levels of social engagement in weight loss and the degree to which support from others was helpful for motivation (see Maitland [18] for previous work on social factors relevant for weight loss). Participants differed in how much they cared about having a social network involved in their weight loss efforts and whether it consisted of strangers, acquaintances, or friends. While some embraced the opportunity to feel a sense of community and having others involved with their weight management efforts, many were also less interested. This ranged from some participants who were ambivalent, to others who were adamant that they were not interested in other people’s diets at all.

About half of the participants mentioned using social support to motivate weight loss. This included using friends, support groups, partners, and nutritionists: “My hubby, I always rush back to tell him if I’ve lost some weight, and he’ll be very encouraging” [P5] and

(My girlfriend) was very supportive, very helpful. She’s super thin by genetics. But when we’d go out she’d order very little food like I would, just so she wouldn’t be eating a giant pile of food while I was restricting myself. Later on, she’d eat more only when I wasn’t around.

P8

Several participants said they derived a sense of connection from others in similar situations: “It is really useful, when you read other people’s stories and what inspired them…and those stories always do keep you going” [P4] and “Who cares that I ate a sandwich with carrots in it…these people do! Guess what I had at lunch, a carrot sandwich. It’s nice to share the journey” [P5].

P6 spoke of the strength she gained from having group support: “this group, we are all people with the same anguish…and this is what makes the group strong, we have the same goal, we have same problem” [P6].

In contrast, some participants were also very uninterested in sharing their weight loss journey with others. They felt that weight management was a private issue and that it did not matter what others were doing. When asked whether they were hearing about other people’s weight management stories, P7 said: “Not really…I’m not interested in that trivial aspect of people’s life. I find it intrusive”. When asked if it would help to know other people are struggling too, P7 replied “Totally irrelevant”.

Several participants mentioned that seeing others’ progress could inspire a sense of motivating competition: “It was amazing how competitive I got with my cousin in this game…if I saw her losing 2 pounds I would be desperate to one up her” [P7].

On the other hand, others distinctly disliked the idea of competing with others about weight loss and felt it would hinder them: “It would hinder me. I would feel demoralized, I’m the kind of person whom if I feel like if I’m competition, I refuse to compete” [P3].

##### Encouraging Messages

Similarly, there were divergent opinions on how helpful motivating messages were, both from other people and from technology applications. While the majority of participants felt motivating messages either from friends or devices would not hurt and could possibly help (ie, be rewarding), others felt messages could be a serious annoyance (ie, costly). In particular, 3 participants explicitly mentioned feelings of annoyance at receiving general motivational feedback and messages. For example, when P8 was asked how he would feel about receiving general motivational messages through an application, he replied:

I think I’d be open to it if they were funny, or just light. It can be kind of annoying, there’s a fine line between being fun and inspirational and being just…annoying. If someone is too encouraging to you, then I feel like it starts to become a negative…when I feel the motivation starts to become someone else trying to impose their schedule on me, or their goals on me then I become resistant to it.

P8

Similarly, P3 spoke of the annoyance of having his motivation externalized and of receiving undeserved congratulations:

It’s just annoying because they are valuing my pathetic (attempts towards goals), it’s patronizing. It’s like you’re not actually seeing when I have done a great job or not. And I also don’t like people externalizing my reward…it makes me feel like I’m jumping through hoops like a trained dog.

P3

P13 expressed annoyance at receiving generic messages from her mobile application that she felt did not address her personal struggles:

A computer telling me, “gain 15 lose 10…Good job I met my goals at the end of the day”—I couldn’t care less. I need it to be personal on some level, and if it’s not, and the computer says “good job”, I’m like “f*** off, who are you to tell me that? You’re just a machine, I don’t appreciate it”.

P13

Thus, while encouragement is usually perceived as a positive motivating factor, our results show that motivational messages can have the opposite effect.

### Quantitative Study Results

#### Summary

We consolidated the themes from our qualitative study into statements in a Web-based questionnaire and asked 100 respondents to rate on a 7-point scale their agreement with each of our statements. Of our respondents, 56 were trying to lose weight (median BMI 26.6), 20 were trying to maintain weight with a focus on not gaining (median BMI 23.5), 5 were trying to maintain weight with a focus on not losing (median BMI 21.6), 8 were trying to maintain weight with a focus on not gaining or losing (median BMI 20.7), 5 were trying to gain weight (median BMI 21), and 6 did not think about their weight (median BMI 21.6). For those who were trying to lose weight, the median reported target weight loss was 30 pounds. We limit analysis to the responses from those who reported wanting to either lose or maintain weight with a focus on not gaining as they are the population relevant for our study, leaving us with 76 participants for our analysis. Of the 56 participants who reported wanting to lose weight, 27 were female. Of the 20 participants who reported wanting to maintain weight with a focus on not gaining, 9 were female.

We assessed the degree of agreement over perspectives and experiences by segmenting our participants into those who agreed (reported slightly, moderately, or strongly agreeing with the statements), the proportion of participants who were neutral (reported neither agreeing nor disagreeing) and the proportion who disagreed (reported slightly, moderately, or strongly disagreed). We arrange our presentation of results into constructs that discouraged and encouraged weight management.

#### Discouraging Constructs

We assessed agreement with the following statements that represented constructs that we found in our qualitative study to be discouraging for weight management efforts:

- I find weight management requires a lot of effort.
- I find the process of denying myself foods I find pleasurable a difficult aspect of weight management.
- It is difficult to keep to my diet in social situations because I want to be able to eat and drink like everyone else.
- I find planning ahead for weight management difficult.
- I find the long-term commitment required for successful weight management daunting.
- I find the process of trying to lose weight a slow and frustrating process.
- I find the notion of being on a diet unappealing.
- I find maintaining a regular exercise routine is difficult.

**Figure 1:**
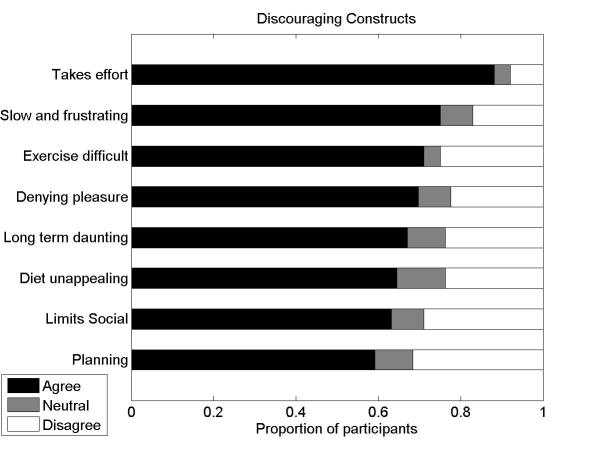
The proportion of respondents who agreed, were neutral, or disagreed with statements reflecting constructs that discouraged successful weight management.

For all discouraging constructs, there was high agreement among participants and all constructs had reported agreement of at least 59% (45/76). The idea that weight management takes a lot of effort was the most agreed upon construct (see Figure 1).

#### Encouraging Constructs

We assessed agreement with the following statements that represented constructs that our qualitative study found were encouraging for weight management efforts:

- I find weight management becomes easier with time.
- I experience feelings of pride surrounding my weight management efforts.
- I enjoy feeling in control when I make choices that are positive for managing my weight.
- I find that it was important to find a weight management strategy that is specifically suited to my individual tastes and lifestyle preferences.
- Being realistic is a useful concept for my weight management.
- Being good to oneself is a useful concept for my weight management.
- Support from other people helps me a lot in my weight management.

**Figure 2:**
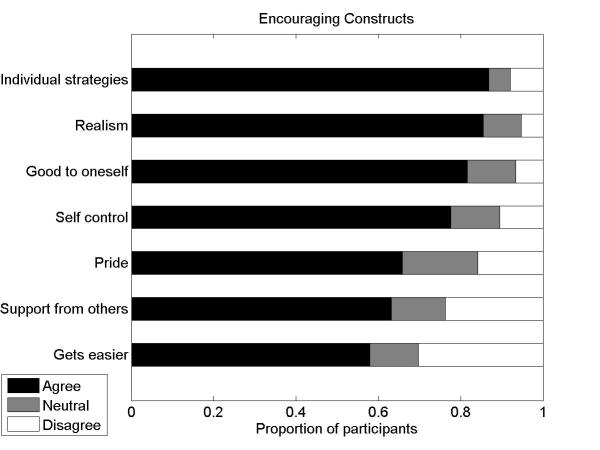
The proportion of respondents who agreed, were neutral, or disagreed with statements reflecting constructs that encouraged successful weight management.

For all encouraging constructs, there was over 58% (44/76) agreement. The most agreed upon constructs were that finding individually tailored strategies was important, the importance of realism, being good to oneself, and the enjoyment of being in control. The most disagreed upon constructs were that it gets easier with time and support from others was helpful (see Figure 2).

#### Social Motivation

We had noticed a greater range of disagreement from our qualitative interviews over how much motivation from others was helpful. Thus we probed this in greater detail. We asked whether people enjoyed hearing others’ stories in person and online, whether people enjoyed receiving motivational messages either in person or online, and whether people benefited from a sense of competition. We asked participants to rate agreement with the following statements:

- I would enjoy reading about other people’s weight management stories *via the Internet*.
- I would enjoy hearing about other people’s weight management stories *in person*.
- I would enjoy receiving motivational messages from others *via the Internet* to help encourage my weight management efforts.
- I would enjoy receiving motivational messages from a computer-based program to help encourage my weight management efforts.
- I would enjoy receiving motivational messages from others *in person* to help encourage my weight management efforts.
- I find a feeling of competition motivating for weight management.

**Figure 3:**
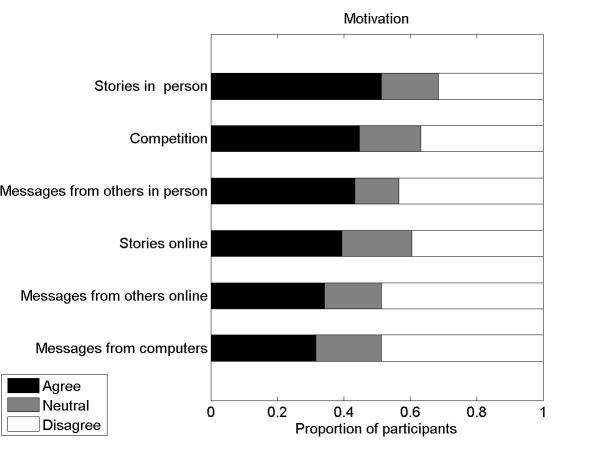
The proportion of respondents who agreed, were neutral, or disagreed with statements reflecting different ways and modes by which one could be motivated by others for weight management.

For factors of external social motivation, we found there was substantially greater disagreement, especially in comparison to the relatively high agreement we had in response to other statements. None of the detailed statements about external social motivation received greater than 51% (39/76) agreement (see Figure 3). Motivational messages and stories received via the Internet were perceived as less helpful than those received in person. Only 32% (24/76) of respondents said they would enjoy motivational messages delivered from a computer, and 49% (37/76) of respondents reported that they would not enjoy this. These results correspond to the variability in attitudes that we found in our qualitative study results regarding motivational messages from others, and also dislike for computer-based motivational messages.

## Discussion

### Principal Results: Rewards, Costs, and Strategies

The themes from our study highlight the many rewards and costs that influence both positive and negative weight management behavior. Our studies found that a key difficulty of weight management was the loss of rewards associated with unhealthy behaviors, such as uninhibited consumption and ability to participate freely in social situations. There were also directly experienced costs associated with weight management. A central cost was the effort required to restrict consumption, change habits, and overcome social pressures. Other costs included negative feelings such as dislike for the concept of dieting, disheartenment at the idea that efforts need to be sustained long term, and frustration when weight loss was slow and there was no perceptible progress. On the other hand, our interviews showed that there are a variety of rewards associated with positive weight management behaviors. These included feelings of pride, self-control, pleasure in taking care of oneself, and enjoying delicious healthy foods. There were also costs associated with unhealthy behaviors such as feelings of guilt, sadness, and low self-esteem. In addition to relevant rewards and costs, our study also revealed commonly used strategies to motivate weight loss efforts. Most universally, participants mentioned finding ways to reduce the cost of weight loss efforts by seeking personalized strategies that addressed individual lifestyles and preferences, and some also felt that weight management gets easier with time. Participants also mentioned the benefits of using social support, and receiving encouraging messages (although as mentioned above, there were significant individual differences in responses to support and encouragement, which is consistent with previous work [18,40]). Finally, participants motivated weight management through emphasizing awareness and realism and reminding themselves of the negative consequences of unhealthy behavior.

**Figure 4:**
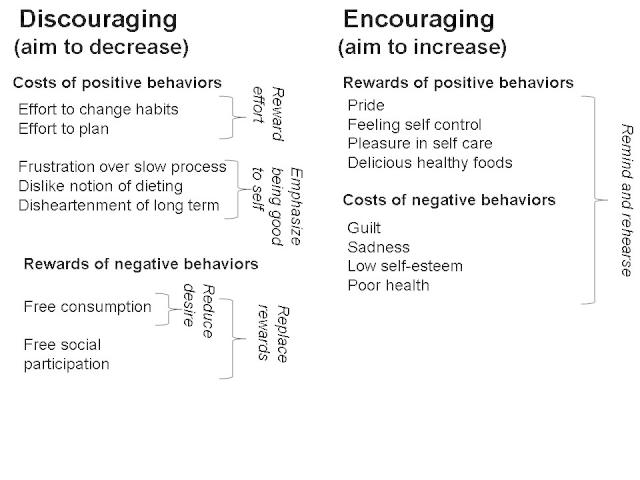
A summary of psychological constructs relevant to weight management.

The psychological constructs identified in our study are summarized in Figure 4. They are organized into those that encourage weight management, which need to be increased (the rewards of positive behaviors and the costs of negative ones), and those that discourage weight management, which need to be lessened (the costs of positive behaviors and the rewards of negative ones). On the side, in italics, are shown general design principles that can be used to increase/decrease the rewards and costs in the appropriate direction to effect positive behavior change.

### Design Guidelines

#### Overview

The themes uncovered in our study reveal a variety of costs and rewards associated with weight management behaviors as well as a range of strategies people used to manage their behavior. As mentioned above, decision theory suggests that healthy behaviors can be encouraged by enhancing the associated rewards and decreasing the associated costs. Similarly, unhealthy behaviors can be discouraged by decreasing the associated rewards and increasing associated costs. We apply these principles to our study results to produce design guidelines for weight management systems, summarized in Figure 4. Below we discuss these principles in more detail. We review how they have been implemented in current technologies, and point out where there are gaps that leave open future design opportunities. We also discuss how the constructs in our study can be used for personalization of weight management technology.

#### Decreasing the Costs of Healthy: Reward Effort and Focus on Being Good to Self

Our findings reveal that effort (eg, planning, restricting food, changing habits and exercise) was the main cost that served as an obstacle to weight loss. One way to address this is for technologies to acknowledge the cost of effort by delivering rewards that offset the cost. Mobile technologies have the advantage of being able to act in the moment of need [21]. This provides an opportunity to design systems that can identify moments of unpleasant effort and then understand the types of rewards that will be appropriate to offset the costs and thus encourage effortful behavior. While there has been a significant rise in the development of systems for sensing user states [22], the accuracy of these systems for identifying user-relevant mental states, such as those found in our study, is yet to be verified. Furthermore, there is relatively little work on how to deliver the appropriate intervention once a “costly moment” has been identified. Systems can offset costs by administering rewards directly, or prompting users to seek appropriate rewards through reminders and suggestions. For example, our study found that planning was considered a significant challenge in weight management. A system can support the difficulty of planning, by acknowledging that planning is difficult, and offering rewards such as encouraging messages or virtual points when a user enters weight loss activities into their calendar.

Other costs found in our study were negative feelings such as dislike of the notion of dieting, frustrations over the slowness of weight loss, and disheartenment by the long-term lifestyle change required. One possibility for reducing these costs is to create systems that focus on being good to oneself and self-care (another theme from our study) rather than focus on the goal of losing weight. Indeed, studies on health messages found that those messages that promoted healthy behavior changes without reference to body weight were most effective and well received [41]. Thus, there is reason to believe that it may be useful to create weight management technologies that frame healthy choices as a process of positive self-care rather than a process of trying to lose weight.

#### Decreasing Rewards of Unhealthy: Replace Lost Rewards and Reduce Desire

Another type of “cost” of weight management is the loss of rewards associated with unhealthy behavior. Two strategies for alleviating this are to find healthy replacement rewards and reduce the salience of the unhealthy reward. Our study found, unsurprisingly, that a key loss was the pleasure of unrestricted eating. A very common weight loss strategy is to seek replacement rewards, and this has indeed been implemented in current systems. However, a less explored avenue is to attempt to lessen the perceived reward. Laboratory studies have shown that operant conditioning can reduce people’s interest in unhealthy but tempting foods [42]. It is possible that an application devoted to reducing the desirability of one’s favorite unhealthy foods may be useful. Also, as mentioned above, laboratory research has found methods of reducing food cravings through imagery tasks [32]. While effectiveness has been suggested in laboratory studies, methods for reducing the desirability of unhealthy foods have been little explored in system designs. A second reward associated with unhealthy behavior was the ability to participate freely in social situations. Our participants reported that group meals were challenging for weight control because they felt pressure both from themselves and others to belong by partaking in the same types of (often unhealthy) consumption as others. Our participants also spoke of the importance of not feeling deprived during weight loss. This understanding of rewards and costs leads to a possible role technology can play to alleviate the challenge of not wanting to feel social alienation: When a social activity is scheduled in the calendar, technology can suggest alternative strategies for maintaining social connection even when not sharing consumption, such as a reminder to “focus on finding out something new about someone”, or “ask someone about a hobby they are interested in”.

#### Enhancing Rewards of Healthy: Remind and Rehearse

Participants reported a variety of rewards associated with weight loss. This begs the question of how persuasive systems can be designed to emphasize and enhance these rewards. This is a particularly promising route because research has found that users tend to respond better to systems that persuade through positive rather than negative feedback [16]. Furthermore, laboratory research has found that emphasizing rewards can promote healthier food preferences. Remembering enjoyment of healthy foods results in these foods being chosen more often in later meals [31], and rehearsing memories of a meal reduces later snack consumption [43]. Despite these promising laboratory results, there have been few systems designed specifically to enhance memories of healthy experiences. Technology has the capacity to strengthen memory of rewards through reminders and also prompting users to mentally rehearse positive past experiences. This can be implemented through e-journals, using voice, pictures, or text, and structured to encourage individuals to remember/take note of the good feelings (eg, pride and self-control) associated with healthy choices. These platforms can also encourage people to relish their appreciation of healthy foods. While there have been the beginnings of technologies designed specifically to encourage reflection and awareness [16,17], there is much room for further developments in this area. In particular, to our knowledge, there have been no platforms devoted specifically to celebrating the pleasures associated with healthy choices and little research into how to design systems that best enhance the rewards of healthy choices. This presents open design challenges and opportunities.

#### Enhancing the Costs of Unhealthy: Remind and Rehearse

In additional to acknowledging the long-term consequences for health, many participants also described immediate costs of unhealthy behaviors, including feelings of sadness, guilt, and anger. We found that some participants tried to remind themselves of these negative consequences to dissuade themselves from unhealthy choices. Furthermore, most participants said that in talking through their past experiences during the interview, they achieved new awareness of the negative experiences that resulted from unhealthy choices. This suggests that technologies can be designed to support further awareness and encourage reflection about the costs involved in weight management. Traditional calorie monitors already achieve this at the level of dietary intake [9,11-14]. However, just as systems may enhance awareness of the rewards of weight management, there is also scope for new technologies to be designed to increase awareness of the costs of unhealthy choices. The delivery of health-warning messages has been found to be effective for smoking cessation [44], although it has been studied less in the domain for weight loss. In addition to warnings about health, systems can be designed to help people remember the bad feelings they experienced after the last time they made unhealthy choices. To our knowledge, no such systems have been studied. Here methods similar to those used to enhance the rewards of healthy behaviors (reminding and prompting mental rehearsals) can be used to enhance the costs of unhealthy behaviors. Note that a system designed to promote awareness of costs is different from a system that punishes and delivers negative feedback, which has been found to put off users [16]. Instead, encouraging awareness of costs is consistent with research showing that there are individual differences in motivational preferences: some tend to be motivated by seeking positive gains (promotion focused) and others by avoiding negative consequences (prevention focused) [45-47]. This suggests there is room in the design space for systems that help users increase awareness and memory of negative feelings, as well as the long-term negative consequences that they may experience as a result of unhealthy choices.

#### Personalized Solutions

When it comes to behavioral change, clearly one size does not fit all. The importance of individual preferences was one of the most highly agreed upon constructs in our survey study. Systems should be designed both to contain personalizable features and also to encourage users to seek personalized solutions. The overwhelming majority of our participants who had successfully lost weight spoke of the importance of individually tailored strategies. The effectiveness of personalization has been supported by research on health-messaging where a meta-analysis has found that individual tailoring of information delivered via the Internet improved effectiveness of nutrition interventions [48]. Also, simple text message-based weight loss intervention personalized to individual participants has been shown to be effective [49]. Personalized e-feedback has also been found to increase program adherence [50]. By highlighting the rewards and costs surrounding weight management, we offer further opportunities for personalization. These can serve as a catalogue of psychological constructs used to identify individual differences in weight management and thus guide personalization. Because there are limits to how many changes people are able to focus on at once, one possibility is to characterize an individual by having them indicate a few rewards and costs, which they find to be most salient (eg, someone who especially finds planning difficult or does not like the idea of dieting). A personalizable system can then suggest interventions that focus on these salient factors. Additionally, a catalogue of psychological factors can be used as a method for characterizing the features of existing weight loss technologies, which can be used to help individuals choose systems that support their specific needs.

Finally, personalization is important for features that involve motivation from others. Our study found significant variation as to whether individuals found social support and encouraging messages motivating or seriously annoying. Thus, motivating messages should be administered with caution because they can be hindering as well as helpful.

### Limitations

The themes of our study were based on interviews with relatively well-educated participants from the United Kingdom and United States. While our themes are not obviously related to education or economic status, it is possible that less educated populations or those from different socioeconomic backgrounds or cultures may have different considerations for weight management. For example, recent studies have found links between traits of self control and educational attainment and wealth [46,51]. Thus, it is possible that lower income and less educated populations may need greater self control aids than those from our study.

Finally, our study is based on self-reports and studies have shown that people are not always accurate at making choices that would be best for themselves [52]. Our studies are based on people’s reflections of past experiences, and there may be discrepancies between what people report to be motivating and discouraging factors versus what factors actually are influential in the moment of experience [53]. For example, half our survey respondents reported they would not like to receive motivational messages from a computer. However, previous research has found that computer-based motivational messages during a computer-based task improved people’s positive affect, enjoyment, willingness to work, and self-perceived performance by a factor of 1.5-2 [54]. Remarkably, this improvement occurred even though people knew that these messages were not contingent on their actual performance. While the study [54] was not in the domain of weight loss, this unintuitive result suggests the possibility that computer-based motivation might be more effective than people assume it to be.

### Conclusions

We have shown how a reward-cost driven inquiry is useful for understanding the psychological constructs in a given behavior change context and for informing persuasive systems design. We applied this framework to understanding the psychological constructs that surrounded individuals’ weight management efforts. Our study identifies a variety of rewarding and costly factors that surround individuals’ positive and negative weight management choices. In a follow-up questionnaire study, we found these factors to be experienced by the majority across a wider population. We applied decision theory to these factors to produce suggested design principles for persuasive systems and discuss where these point to open design opportunities that warrant further exploration.

## Multimedia Appendix 1

Qualitative interview survey materials.
