# Supplementary material for: Designing for Psychological Change: Individuals’ Reward and Cost Valuations in Weight Management
Source: J Med Internet Res. 2014 Jun 26;16(6):e138. doi: 10.2196/jmir.3009 (PMC4090383; doi:10.2196/jmir.3009)
Supplement: Supplementary file 1 [file jmir_v16i6e138_app1.pdf]

Multimedia Appendix 1: Instrument used as the basis for semi-structured interviews

Age:
Gender:
Weight:
Height:

-What are your weight goals? Do you have a certain weight you want to be or are you just looking to maintain your weight?

-Time spent on weight goal?

-What is your weight history? Steadily increasing through life or up and down?

-How easy are you finding this goal to achieve?

-What strategies are you doing to achieve these goals? Eg. diet, exercise.

- How easy are you finding these strategies to stick to?

- Think back to something recently you did that helped towards achieving your weight goal, what was it?

- What factors helped? Specifically:
- Emotions
- Thoughts
- Actions
- Situation

-Are there times where it has been easier to achieve your goals? When and why?

-Think back to something that hindered your weight goal, what was it?

- What factors do you feel hindered your goal? Specifically:
- Emotions
- Thoughts
- Actions
- Situation

-Talking about your relationship to food:
- When?- e.g. 3 big meals / snacking
- Where?- e.g. round table / on the go / in front of tv
- How do you eat? - e.g. Social / private eating? Big meals / snacks?

-Does self-image affect your weight goals?

-Does health affect your weight goals?

-What are the triggers for food related thoughts and actions? Certain times of day, activities, feelings?

- Do you have and use regularly the following:
- a smartphone (iphone / android)
- laptop
- ipad or other tablet device

- Do you use apps or websites to aid your weightloss goals?

-Which ones, how do they help you?

- Could you give me an example of a time this app or using your phone normally has helped with your weight goal?

We are looking to develop an app for a smartphone/tablet devise that would help people develop a habit of greater motivation for weight goals. What kind of support/feedback/ (reminders (images/text? Personalized/stock?) would you find helpful for your weight goals?

-How do you feel about an app that prompts you throughout the day vs. an app where you use it when you'd like?

-How about information about other's weight/progress/strategy?

-Do you use mobile apps in general? Which ones do you use and how do you use them?
